# Discovery of Late Triassic bivalves from Jurassic deep-water deposits in Riganpeicuo area, Tibet and their geological significance

**DOI:** 10.1038/s41598-022-12338-7

**Published:** 2022-05-18

**Authors:** Hongji Xiao, Shenglong Luo, Jinhan Gao, Genhou Wang

**Affiliations:** 1 School of Earth Sciences and Resources, China University of Geosciences, Beijing, 100083 China; 2 School of Earth Sciences, East China University of Technology, Nanchang, 330013 China

**Keywords:** Environmental sciences, Ocean sciences, Solid Earth sciences

## Abstract

The Jurassic sequences in the South Qiangtang Basin of Tibet are essential for understanding the paleogeography and tectonic evolution of this basin and the Bangong-Nujiang Meso-Tethys Ocean (BNMO). However, the partial absence of fossils hinders the study of the stratigraphic distribution and tectonic paleogeography of the basin. Late Triassic bivalves, including nine species in nine genera, were identified for the first time in olistostromes from the Sêwa Formation in the Riganpeicuo area. Based on detailed geological field surveys and sedimentary facies analysis, the lower-middle part of the Rigenco section is a deep-water turbidite fan deposit and the upper part is a shallow sea mixed shelf deposit, which differs distinctively from the carbonate platform facies of the Jiebuqu Formation. Therefore, the strata formerly assigned to the Jiebuqu Formation (Rigenco section) is formally reassigned to the Lower-Middle Jurassic Sêwa Formation and Middle Jurassic Shaqiaomu Formation. These results not only provide further understanding of the sedimentary infill history of the South Qiangtang Basin, but also further support that the BNMO was already open in the Late Triassic and formed a mature ocean in the Early-Middle Jurassic, with subsequent rapid subduction. Both the opening and subduction processes were completed in a relatively short time interval with a large number of attendant olistostromes.

## Introduction

Known as the roof of the world, the Qinghai-Tibet Plateau is the most extensive plateau in China and the highest plateau in the world. It also has some of the world’s most active tectonic belts. The Bangong-Nujiang suture zone (BNS), which lies between the Qiangtang block and the Lhasa block, is a relic of the closure and cessation of the Bangong-Nujiang Meso-Tethys Ocean (BNMO). This zone has great geological significance for studying major geological processes, such as the tectonic evolution of the BNMO, the initiation of rifting of the northern margin of Gondwana and the development of the Asian plate^1–4^.

Geological questions surrounding the BNMO, such as the nature, evolution model, subduction polarity, and closure mechanism of the BNMO, have not been resolved to date, and aspects of its evolutionary history remain uncertain. For example, numerous studies have suggested a diverse spread of opening times for the BNMO: Late Carboniferous to Early Permian^5,6^, Early-Middle Permian^7–10^, Late Permian^11,12^, Late Permian to Early Triassic^13^, Late Triassic^14,15^, Early Jurassic^16–19^, and Middle Jurassic^20^, among others, have been proposed. On the other hand, on the issue of the closure time most researchers agreed that the closure of the BNMO happened in the Late Jurassic to Early Cretaceous^7,11,14,21–28^; while some proposed Late Cretaceous^10,17,29^. The uncertainty of the opening and closure time is ascribed to the different studying locations within the South Qiangtang Basin, where the sedimentary sections are different with varying exposure and preservation potential and poor stratigraphic control. The nature of top and bottom boundary are still unknown, resulting in differences in identification of tectonic cycle.

The Riganpeicuo area is located in the central South Qiangtang Basin of the Qinghai-Tibet Plateau, adjacent to the BNS. It is the largest exposed area of Mesozoic sequences in the South Qiangtang Basin and develops with complete stratigraphic sections. These sections provide insight into the characteristics of the tectonic and sedimentary history of the BNMO and the South Qiangtang Basin. However, the lack of fossils and radiometric ages have seriously affected the temporal resolution of sequence stratigraphy, and have also become an obstacle to precise understanding of the structure and evolution of the South Qiangtang Basin and the BNMO.

A large number of bivalve fossils have been found for the first time of this study in the Rigenco section (strata that were previously assigned to the Middle Jiebuqu Formation) in the Riganpeicuo area. The fossils occur within olistostromes, which are inconsistent with the sedimentary characteristics of the Jiebuqu Formation. Therefore, detailed petrological, sedimentological and paleontology studies were carried out to complete the regional stratigraphic and palaeontological data. The main stages of the tectonic evolution of the BNMO are discussed with great implications for understanding its evolution from the opening to closure.

## Geological background

**Figure 1:**
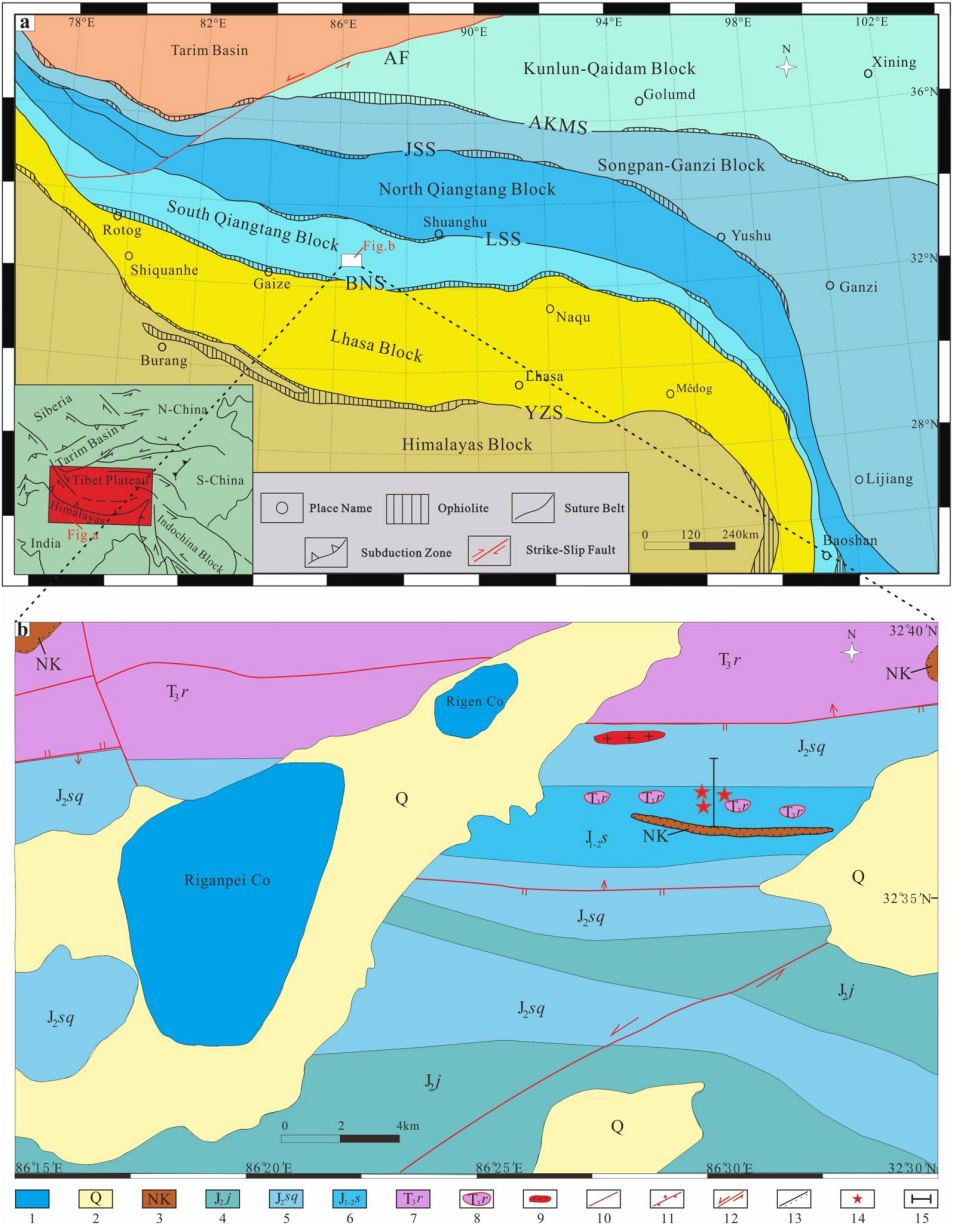
(**a**) Tectonic outline of the Himalaya-Tibetan Plateau showing major terranes and suture zones^15^. (**b**) Geological map of the study area showing the Rigenco section and sample locations^82^. YZS, Yarlung-Zangbo suture zone; BNS, Bangong-Nujiang suture zone; LSS, Lungmu Co-Shuanghu suture zone; JSS, Jinsha suture zone; AKMS, Ayimaqin-Kunlun-Mutztagh suture zone; AF, Arkin Fracture zone; 1, Lake; 2, Quaternary strata; 3, Neogene Kangtog Formation; 4, Middle Jurassic Jiebuqu Formation; 5, Middle Jurassic Shaqiaomu Formation; 6, Lower-Middle Jurassic Sêwa Formation; 7, Upper Triassic Riganpeicuo Formation; 8, Olistostromes; 9, Granite Porphyry; 10, Fault; 11, Reverse Fault; 12, Strike Slip Fault; 13, Angular unconformity; 14, Sampling Points; 15, Location of Section.

The Qinghai-Tibet Plateau is the east part of the Tethys tectonic domain. This plateau is likely the result of the northward movement of blocks formed by multiple breakups of the northern margin of the Gondwanan continent to the south and the sequential collision of these blocks with the Laurasia continent to the north. From north to south, the Qinghai-Tibet Plateau is composed of five blocks: Kunlun-Qaidam, Songpan-Ganzi, Qiangtang, Lhasa and Himalaya; these blocks are divided by the Ayimaqin-Kunlun-Mutztagh suture zone (AKMS), Jinsha suture zone (JSS), BNS and Yarlung-Zangbo suture zone (YZS)^2,3,30–32^ (Fig. 1a). The cold-water *Eurydesma* fauna unique to Gondwana were found in the Zhanjin Formation of Rutog County which was accompanied by a glacial marine gravelly slate^33^. This discovery prompted researchers to define a new northern Gondwanan boundary, namely, the Lungmu Co-Shuanghu suture zone (LSS), and divide the Qiangtang Basin into the North Qiangtang Basin and South Qiangtang Basin^34,35^.

The study area is located in the southern margin of the South Qiangtang Basin and is close to the northern middle segment of the BNS. This area is vital for understanding the tectonic evolution of the South Qiangtang Basin and the BNS. The strata in the study area are part of the Yunnan-Tibet stratigraphic region, Qiangnan-Baoshan strata division, and Duoma subdivision, which mainly contain the Upper Triassic Riganpeicuo Formation (T_3_*r*), Lower-Middle Jurassic Sêwa Formation (J_1–2_* s*), Middle Jurassic Shaqiaomu Formation (J_2_*sq*), Middle Jurassic Jiebuqu Formation (J_2_*bq*), Neogene Kangtuo Formation (N*k*),  and Quaternary strata (Q) (Fig. 1b).

## Materials and analytical methods

The Rigenco section in the northeastern Riganpeicuo area was measured, and the strata were studied in detail by integrating research methods from petrology, sedimentology, stratigraphy and paleobiology.

A large number of bivalve fossils were found in the study area for the first time. In total, 164 fossils were collected. After identification, fossils of nine species in nine genera were photographed by stereomicroscope (LEICA M205C). Through biostratigraphic correlation, the age of the fossil assemblage was determined, and then the age of the fossil-bearing strata was determined.

In addition, 36 rock samples were systematically collected and grounded into 25 mm × 50 mm thin sections. The compositions of the particles, matrix and cement were analyzed under a polarizing microscope (LEICA DM4500P). The carbonate rocks, clastic carbonate rocks and clastic rocks developed in the region were classified according to the rock classification schemes of Dunham^36^, Mount^37^ and Wentworth^38^. The layer thickness, rock color, rock type, assemblage characteristics, sedimentary structures, fossils, and field output were analyzed to determine the stratigraphic sedimentary facies.

## Analytical results

### Stratigraphic features

**Figure 2:**
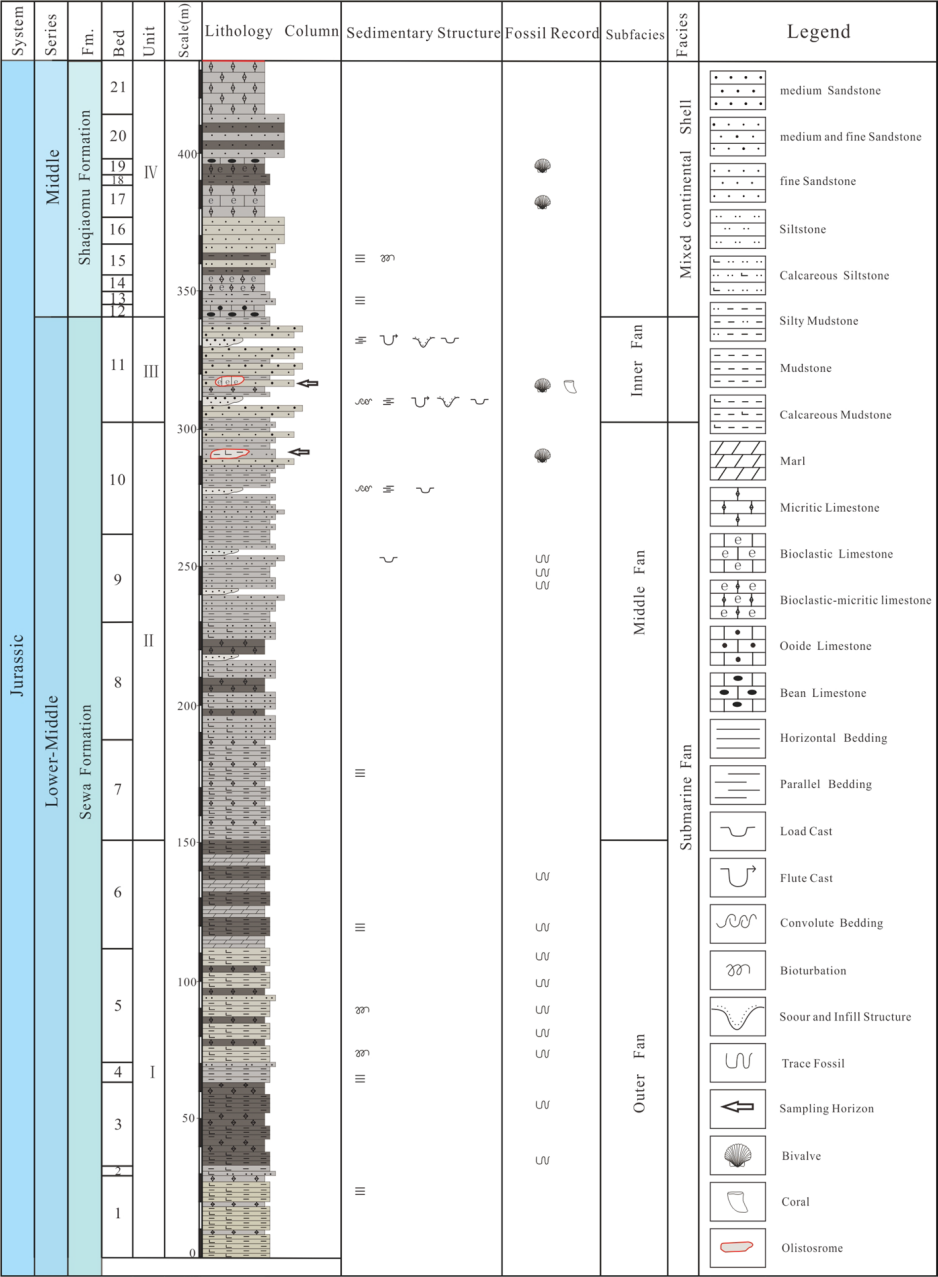
The stratigraphic log of the Rigenco section in the northeastern Riganpeicuo area in the South Qiangtang Basin, Tibet. Fm. = Formation.

The Rigenco section is located in the northeastern Riganpeicuo area, South Qiangtang Basin, Tibet. The coordinates of the profile positions are 32°37′2.12" N and 86°23′44.34" E, and the elevation is 4955 m (Fig. 1b). The section is 435 m in thickness. The base of the measured section is covered by the Neogene Kangtuo Formation, so its contact relationship with the underlying geology cannot be seen. The top of the section is in fault contact with the Upper Triassic Riganpeicuo Formation. Based on lithologic assemblage and sedimentary structure, the Rigenco section can be subdivided into four distinct parts from bottom to top (Fig. 2).

Layers 1–6 (151.3 m thick) contain rhythmic interbedding of gray, gray-black thin to very thin-bedded calcareous mudstone with calcareous siltstone. Horizontal bedding is developed, and numerous trace fossils occur in parallel layers.

Layers 7–10 (151.4 m thick) contain rhythmic interbedding of gray thin-bedded mudstone with siltstone, interbedded with small fine sandstone bodies. Calcareous mudstone gravel and calcareous mudstone olistostromes are visible in the lower part. Horizontal bedding is developed in the mudstone and siltstone. Small-scale cross-bedding, plus cross-bedding and convolute bedding are developed in the fine sandstones, and load cast structures are visible. The siltstone and mudstone yield trace fossils with small angle oblique planes, and the calcareous mudstone olistostromes contain numerous bivalve fossils.

Layer 11 (38.2 m thick) contain gray medium-thick-bedded medium-fine sandstone interbedded with gray thick-bedded medium sandstone and intercalated with gray-black thin-bedded silty mudstone, forming multiple cycles. The bottom is mostly coarse sandstone and partially intercalated with large-scale conglomeratic sandstone lenses along with bioclastic limestone olistoliths of varying sizes and shapes. On the bottom surface of the stable sandstone bed, groove casts, flute casts, and load casts are present. Parallel bedding, large convolute bedding, and graded bedding are visible. Abundant bivalve fossils are present in the limestone olistostromes.

In Layers 12–21 (92.6 m thick) the bottom is interbedded with gray medium-thin-bedded limestone and gray thin-bedded silty mudstone and siltstone. The upper part is interbedded with gray medium-thin limestone and gray-white medium-thick feldspathic quartz fine sandstone and silty mudstone with non-uniform thickness. The limestones mainly include micritic limestones, oolitic limestones and pisolitic limestones. Parallel bedding and cross-bedding are developed in the fine sandstones, and horizontal bedding is developed in the silty mudstones and siltstones.

### Identification of bivalve fossils

**Figure 3:**
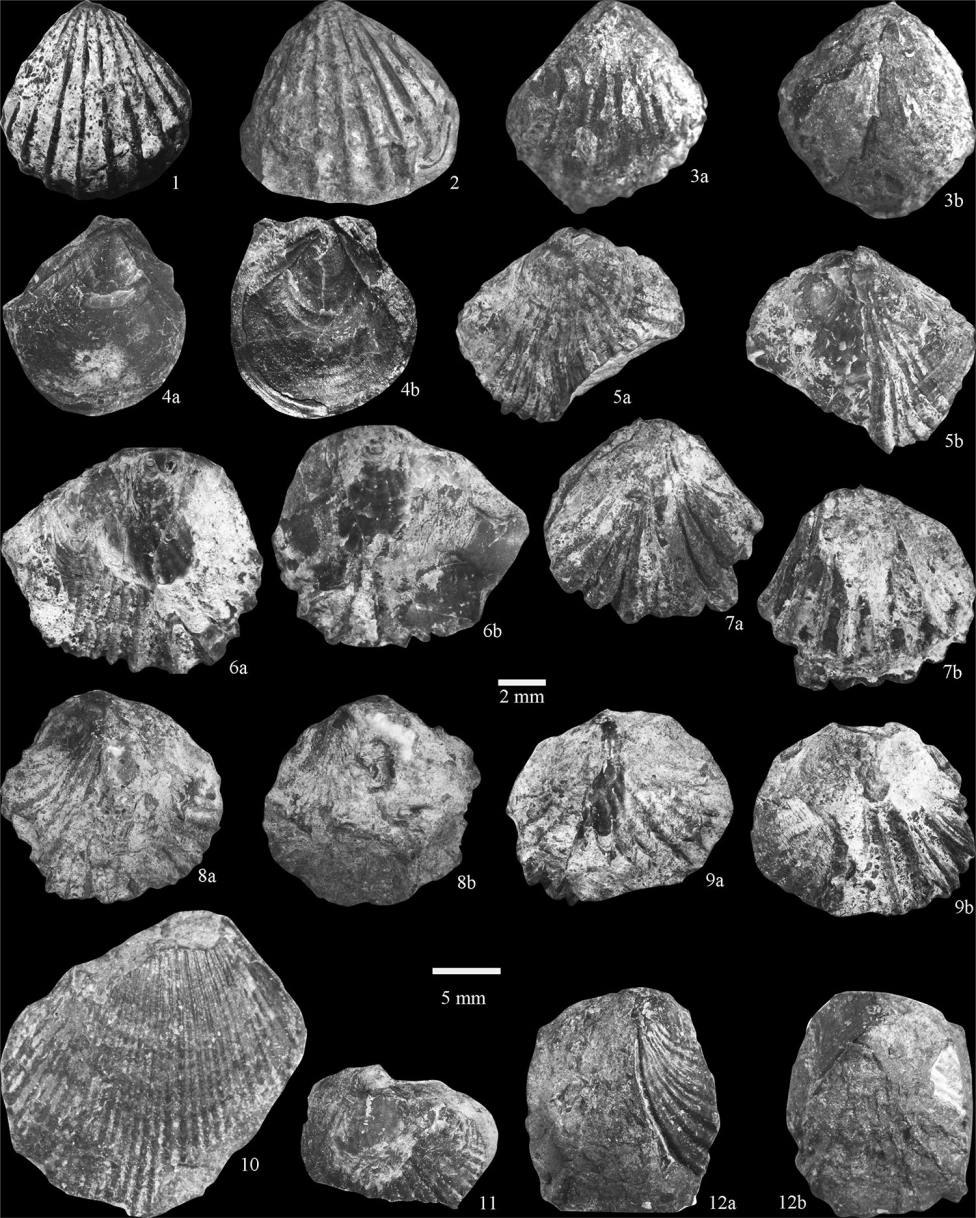
Bivalves of Rigenco Section in the Riganpeicuo area, South Qiangtang Basin, Tibet. 1–3 *Palaeocardita* sp., (1–2, 3b) right valve, (3a) left valve, specimen no. 1–2, 1–4, 1–11; 4 *Entolium fimbriatum*, (4a) right valve, (4b) internal mold of right valve, specimen no. 1–7; 5 *Halobia* sp*.*, (5a) right valve, (5b) left valve, specimen no. 1–6; 6 *Leptochondria* sp., (6a) right valve, (6b) left valve, specimen no. 1–3; 7, 9 *Costatoria* sp*.*, (7a, 9a) right valve, (7b, 9b) left valve, specimen no. 1–27, 3–2; 8 *Lopha* sp. (8a) right valve, (8b) left valve, specimen no. 1–8; 10 *Lima* sp., right valve, specimen no. 6–1; 11 *Burmesia lirata*, left valve, specimen no. 2–2; 12 *Badiotella* sp., (12a) right valve, (12b) left valve, specimen no. 1–28; for (1–7), scale bar = 2 mm; for(8–12), scale bar = 5 mm.

In this study, bivalve fossils were collected from calcareous mudstone olistostromes in layer 10 and bioclastic limestone olistostromes in layer 11 of the Sêwa Formation. But the bivalve fossils from the bioclastic limestone olistostromes in layer 11 are poorly preserved and cannot be identified. From the collected bivalve fossil samples, nine species belonging to nine genera were identified: *Badiotella* sp., *Burmesia* sp., *Costatoria* sp., *Entolium fimbriatum*, *Halobia* sp., *Leptochondria* sp., *Lima* sp., *Lopha* sp., and *Palaeocardita* sp. (Fig. 3). The genera *Burmesia and Palaeocardita* are the most abundant taxa collected (Table 1).

**Table 1:** Species richness of bivalve fossils in the Sêwa Formation of the Rigenco section in the northeastern Riganpeicuo area, Tibet.

Species	Abundance
*Badiotella* sp.	+ + + + +
*Burmesia* sp.	+ + + + + +
*Costatoria* sp.	+ +
*Entolium fimbriatum*	+ +
*Halobia* sp.	+ +
*Leptochondria* sp.	+ + + +
*Lima* sp.	+
*Lopha* sp.	+ +
*Palaeocardita* sp*.*	+ + + + + + +

## Discussion

### Age of the bivalve fossil assemblages

*Badiotella* is a widely distributed genus in the Middle to Upper Triassic strata in China. It has been reported from the Upper Triassic Carnian Sanqiao Formation in Guizhou Province^39^, Carnian Hanwang Formation in the Maantang area, Sichuan Province^40^, Upper Triassic Norian-Rhaetian strata in the Ali area, Tibet^41^, and Ladinian-Rhaetian strata in the Western Tethys^39^.

*Burmesia* is a common genus in the Upper Triassic series in Sichuan, Tibet, Qinghai, Yunnan, Guizhou and other provinces of China. It is found in the Upper Triassic Maantang Formation, the Carnian Kwahongdong and Shemulong Formations, the Norian Xujiahe and Shiyuan Formations in Sichuan Province^42,43^, the Upper Triassic Luojiadashan Formation, the Norian-Rhaetian Huobachong Formation, the Carnian Songgui and Weiyuan Formations in Yunnan Province^42,44^, the Upper Triassic Norian-Rhaetian stage in the Gerake area, Tibet^41^, the Upper Triassic Carnian-Norian Langjiexue Formation in Tibet^45^, the Carnian-Norian Xiaomeidong Formation in Qinghai Province^42^, the Carnian Sanqiao Formation in Guizhou Province^42^. It is also found in Norian strata in Myanmar^46^, Norian strata in Buru and Misol in Indonesia^46^, Norian strata in Vietnam^46,47^, Norian strata in Indochina^42,46^, and Norian strata in Japan^46^.

*Costatoria* is distributed in Triassic strata worldwide^39,48^. It occurs within the Carnian Banan Formation in Guizhou Province^49^, Anisian Mojia Formation in the Gejiu area, Yunnnan Province^50^, Carnian Heimiaowan Formation in the Youjiang Basin, Guangxi Province^51^, Norian Shizhongshan Formation in the Jianchuan area, Yunnnan Province^39^, Anisian-Ladinian Na Khuat Formation in North Vietnam^52^, and Upper Triassic strata in Southwest Japan^53^.

*Entolium* is distributed in Mesozoic strata in Europe, Asia, and North America. It occurs within the Upper Triassic Norian-Rhaetian Huobachong Formation in Yunnan Province^44^, the Carnian Hanwang Formation in the Jiangyou area, Sichuan Province^40^, Upper Triassic Norian-Rhaetian strata in the Gerake area, Tibet^41^, and the Lower Jurassic Pusela Formation in the Ali area, Tibet^54^. *E. fimbriatum* is found in the Upper Triassic strata in the Kaiyuan area, Yunnan Province^39^*.*

*Halobia* is widely distributed in the Middle Triassic to Upper Triassic strata of Europe, Asia, America, and Oceania. It has been reported in the Carnian Mangxiangyu Formation and Norian Gongzula Formation in Ali area, Tibet^54^, the Carnian-Norian Langjiexue Formation in the Tethys and the Himalaya, Tibet^45^, the Carnian Kangshare Formation and Norian Qulonggongba Formation in the Himalaya area, Tibet^55^, the Norian Bolila Formation in the Yushu area, Qinghai^56^, the Upper Triassic Huobachong Formation in Yunnan Province^44^, the Norian Xujiahe Formation in Sichuan Province^43^, the Carnian-Rhaetian strata in the Songpan-Ganzi area, Sichuan Province^39^, the Carnian-Rheatian strata in Japan^48^, the Carian-Norian Pardonet Formation in British Columbia, Canada^57^, the Upper Triassic in North America^48,58^, Carnian-Norian strata in Vietnam^47,59^, the Carnian Mae Thang Formation in Thailand^59^, the Carnian Bagong Formation and Upper Triassic Langjiexue Group in Tibet^45,60^, Carnian-Norian strata in Buru and Misol in Indonesia^61^.

*Leptochondria* is distributed in Triassic strata worldwide^48^. It is found in the Anisian-Ladinian Na Khuat Formation in North Vietnam^52^, Early Triassic Feixianguang Formation in Guizhou Province^62^, and Middle Triassic strata in Nevada, North America^63^.

*Lima* is reported in the Triassic strata in Tibet, Western Tethys, Yunnan, Indochina, and New Zealand^39,41,48^.

*Lopha* is reported in the Triassic strata in Tibet, Western Tethys, South China, and Indochina^48^.

*Palaeocardita* is reported in the Upper Triassic Norian-Rhaetian Huobachong Formation and Carnian Luojiadashan Formation in Yunnan Province^42,44^, the Norian Xiaotangzi Formation in Sichuan Province^40^, the Carnian Resha Formation, Norian Qulonggongba Formation, and Rhaetian Derirong Formation in the Himalaya area, Tibet^55^, the Carnian-Norian Jiapila Formation in the Yushu area, Qinghai Province^56^, the Norian Namyau Group in Myanmar^59^, the Norian Mae Thang Formation in Thailand^46^, the Triassic strata in South America, North America, Indochina, New Zealand, and the Arctic^39^.

According to the stratigraphic distribution of the above bivalve fossils (Table 2), the age of the bivalve assemblage is determined to be Late Triassic. Marine bivalves were reported in the Riganpeicuo Formation, including *Burmesia*, *Costatoria*, *Indopecten*, *Palaeocardita, Pectinacea*, and *Schafhaeutlia*^64,65^. These fossils occur in redeposited sediments (calcareous mudstone olistostromes). Hence, it is reasonable to conclude that the olistostromes are originated from the Riganpeicuo Formation.

**Table 2:**
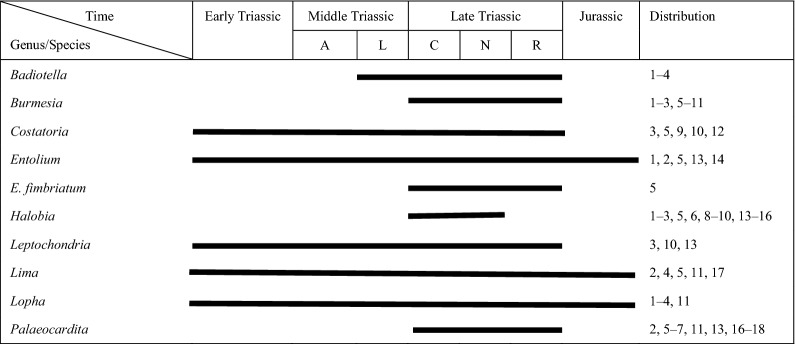
Stratigraphic distribution of bivalves in the Sêwa Formation of the Rigenco section in the northeastern Riganpeicuo area, Tibet.

A = Anisian; L = Ladinian; C = Carnian; N = Norian; R = Rhaetian; 1 = Sichuan Province, China; 2 = Tibet, China; 3 = Guizhou Province, China; 4 = Tethys; 5 = Yunnan Province, China; 6 = Qinghai Province, China; 7 = Myanmar; 8 = Indonesia; 9 = Japan; 10 = Vietnam; 11 = Indochina; 12 = Guangxi Province, China; 13 = North American; 14 = Europe; 15 = Canada; 16 = Thailand; 16 = Iran; 17 = New Zealand; 18 = South American.

### Sedimentary facies of the Rigenco section

**Figure 4:**
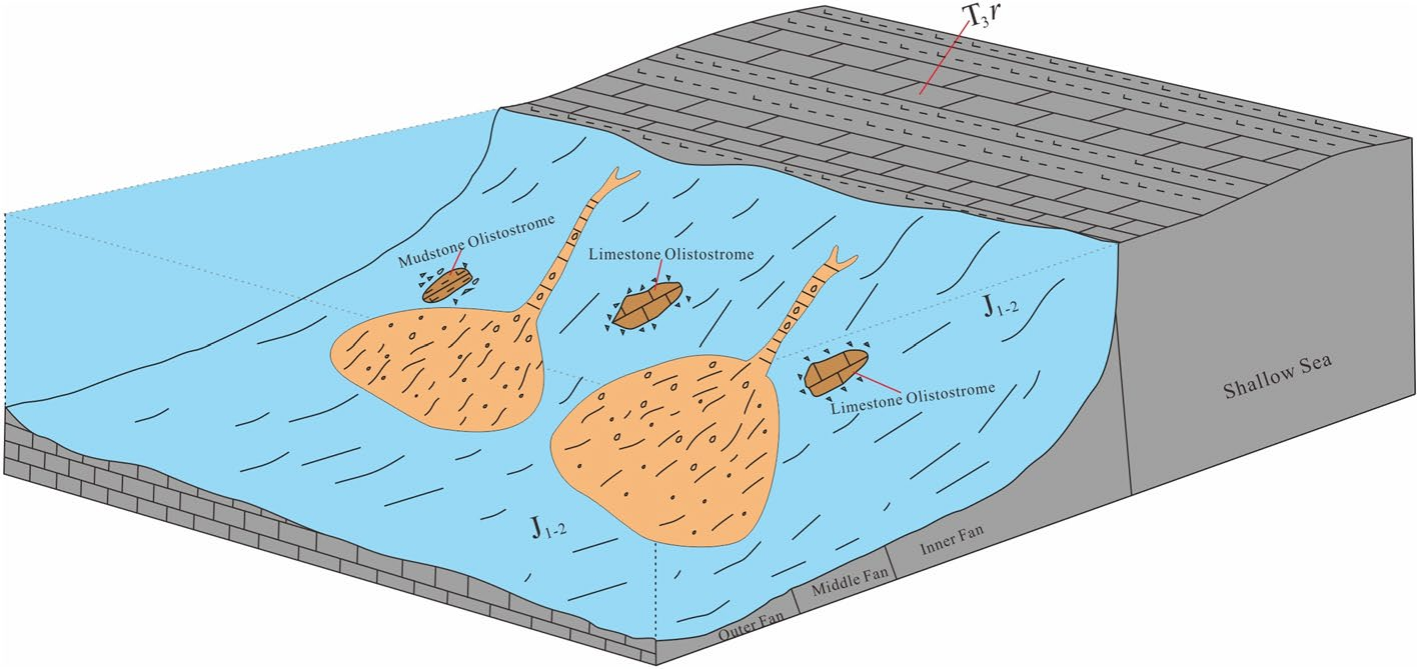
Model chart of the sedimentary facies of the Rigenco section in the northeastern Riganpeicuo area, South Qiangtang Basin, Tibet.

The sedimentary characteristics of the Rigenco section in the study area show that the lower-middle parts (layers 1–11) are a set of turbidite submarine fan deposits, and that the upper parts (layers 12–21) are shallow sea mixed shelf deposits (Fig. 2). The layers are in primary depositional contact. From bottom to top, the submarine fan can be divided into three sedimentary subfacies, namely, the outer fan, middle fan and inner fan (Fig. 4), composing an upward-shallowing progradational sequence.

**Figure 5:**
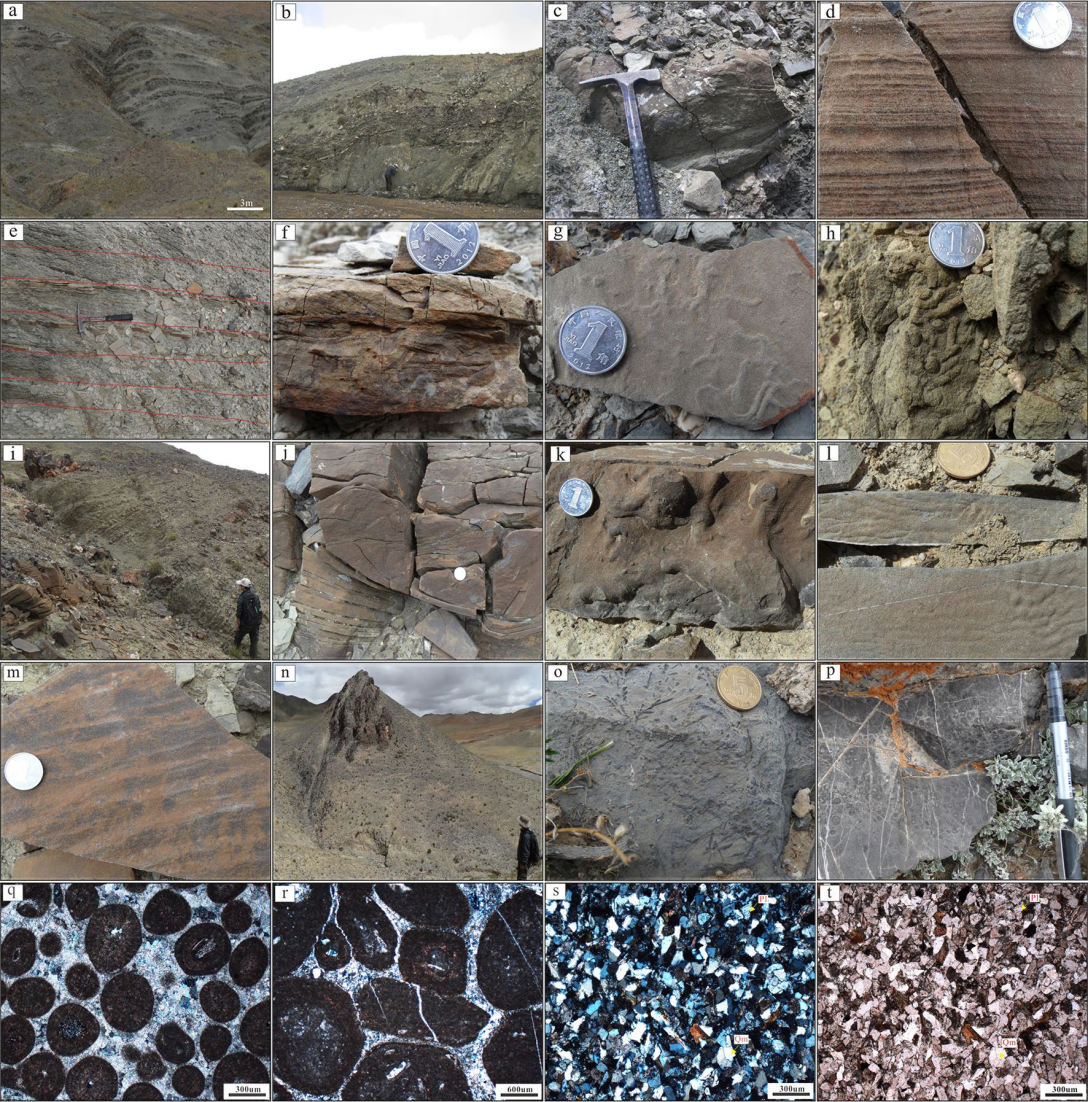
Field photographs and photomicrographs showing the lithologic composition characteristics and sedimentary structures of the Rigenco section in the northeastern Riganpeicuo area, South Qiangtang Basin, Tibet. (**a**) Interbedding of thin bedded mudstone and siltstone (layer 4); (**b**) Calcareous mudstone olistostromes (layer 10); (**c**) Lenticular channel sand body with parallel bedding (layer 9); (**d**) Parallel bedding is developed in the fine sandstone (layer 9); (**e**) Rhythmic interbedding of mudstone and siltstone (layers 7–10); (**f**) Small sand bedding is developed in the sandstone (layer 9); (**g**) Trace fossils of parallel layers are developed in the siltstone (layer 9); (**h**) Trace fossils are developed in the fine sandstone (layer 10); (**i**) Bioclastic limestone olistostromes (layer 11); (**j**) Convolute bedding is developed in the sandstone (layer 11); (**k**) Load cast is developed in the sandstone (layer 11); (**l**) Flute casts and groove mold are developed in the fine sandstone (layer 11); (**m**) Groove mold is developed in the fine sandstone (layer 11); (**n**) Stable layered limestone (layer 17); (**o**) Chondrites are developed in siltstone (layer 15); (**p**) Oolitic limestone (layer 12); (**q**) Oolitic limestone (layer 12); (**r**) Pisolitic limestone (layer 19); (**s**) Fine grained feldspathic quartz sandstone (crossed polarized light, layer 18); (**t**) Fine grained feldspathic quartz sandstone (single polarized light, layer 18).

In the outer fan facies (layers 1–6), segments C, D and E of the Bouma sequence are developed, and there is no evidence of a channelized sand body. This facies are mainly composed of gray to gray-black, thin to very thin calcareous mudstone and siltstone with rhythmic interbedding (Fig. 5a). The thickness of mudstone is greater than that of siltstone within a single rhythmic unit. Locally, siltstone with fine sand cross-bedding in segment C and bulky mudstone in segment E are visible.

The middle fan facies (layers 7–10) are characterized by the development of branching channels and fan-front lobes. There are numerous of bivalve fossils preserved within the large calcareous mudstone olistostromes. The calcareous mudstone olistostromes (Fig. 5b) are lens-shaped, and quite different lithologically from the surrounding rock strata. The main body is composed of segments B, C, D, and E of the Bouma sequence. The branched channels are represented within segment B and are mainly consist of lenticular medium-fine sandstones with variable internal structures: massive bedding, graded bedding and parallel bedding (Fig. 5c, d). The lobes between channels and in front of the fan are made up of segments C, D and E, and the lithology is mainly rhythmically interbedding of thin silty mudstone and siltstone (Fig. 5e), intercalated with sandstone with fine cross-bedding (Fig. 5f) and siltstone. Massive mudstone is locally visible. Numerous trace fossils in parallel layers are developed in the silty mudstone and siltstone (Fig. 5g), and trace fossils in oblique layers are developed in the sandstone (Fig. 5h).

The inner fan facies (layer 11) are characterized by the development of channels and natural sedimentary dikes. There are sizeable bioclastic limestone olistostromes with different dimensions and shapes (Fig. 5i). The main body is composed of segments A, B, C and D. Compared with the middle fan, the inner fan has fewer channels but larger scale. The channel-form sand bodies are represented by segments A and B, which are large lenticular sandstone and glutenite bodies with graded bedding, massive bedding and parallel bedding. The natural levee is composed of segments C and D, which are mainly composed of thin-layer silty mudstone and siltstone with variable thicknesses of interbedding, intercalated with fine sandstone and siltstone with fine cross-bedding. Sedimentary structures such as convolute bedding (Fig. 5j), load casts (Fig. 5k), flute casts (Fig. 5l), and groove casts (Fig. 5l, m) are visible in the sandstone. Numerous trace fossils are developed in the fine siltstone and medium sandstone.

The upper part of the profile is of mixed shelf facies. It is composed of gray stratified limestone (Fig. 5n), such as micritic limestone, oolitic limestone (Fig. 5p, q), pisolitic limestone (Fig. 5r), silty mudstone, siltstone, and fine sandstone with variable interbedding thicknesses. The fine sandstone (Fig. 5s, t) contains cross-bedding and parallel bedding. The siltstone and silty mudstone include horizontal bedding. Trace fossils are developed in the siltstone (Fig. 5o), and bivalve fossil fragments are common.

### Interpretation of the Rigenco section

The Mesozoic marine strata in this area include, from bottom to top, the Upper Triassic Riganpeicuo Formation, Lower-Middle Jurassic Sêwa Formation, Middle Jurassic Shaqiaomu Formation and Jiebuqu Formation. These formations are in primary depositional contact with each other. The Riganpeicuo Formation (T_3_*r*) is a sequence of shallow platform facies carbonate rocks that are rich in fossils of taxa such as hexahedral corals, sponges and bivalves^66^. The Sêwa Formation (J_1–2_* s*) is a sequence of deep-water turbidite fan deposits dominated by deep fine clastic rocks, with a typical Bouma sequence characteristics, graded bedding, various current-related sedimentary structures, and abundant trace fossils^15,67,68^. The Shaqiaomu Formation (J_2_*sq*) is a sequence of gray bioclastic limestones, oolitic limestones, micritic limestones, quartz sandstones and siltstones that represent a mix of reef limestone shelf facies, shallow sea carbonate and terrigenous clastic sedimentary facies, and abundant coral reef facies^67,69^. The Jiebuqu Formation (J_2_*bq*) is a sequence of shallow marine carbonate deposits composed of gray-black, dark gray giant thick-bedded bioclastic limestones, thick-bedded micritic limestones, thick-bedded dolomitic micritic limestones, and limestone breccias^19,68,70–72^.

According to the above biostratigraphic correlations, the ages of the calcareous mudstone olistostromes are constrained to the Late Triassic, so the sedimentary age of the strata in the study area must be younger than Late Triassic. Furthermore, the sedimentary facies analysis shows that the main body contains hemipelagic-pelagic turbidite fan deposists. The upper part is mixed shelf facies with stable carbonate rock and clastic rock interbeds of varying thicknesses. According to the regional stratigraphy, the main body of the section is consistent with the Lower-Middle Jurassic Sêwa Formation, and the upper part (layers 12–21) is consistent with the sedimentary characteristics of the Middle Jurassic Shaqiaomu Formation. Therefore, it is suggested here that the strata which were previously assigned to the Middle Jiebuqu Formation should be reassigned to the Sêwa Formation and the Shaqiaomu Formation.

### The limits of the Bangong-Nujiang Meso-Tethys Ocean

According to the above analysis and regional geology, the Mesozoic geological evolution of the South Qiangtang Basin was primarily controlled by the tectonic evolution of the middle segment of the BNMO, and its basin infill history was the sedimentary response to its tectonic history.

In the Late Triassic, the BNMO opened and entered an initial stage. The marine environment in the South Qiangtang area evolved into a shallow sea with carbonate deposits of the Riganpeicuo Formation (T_3_*r*), as is evident from the fossil record of corals, sponges, bivalves, crinoids, and other shallow sea organisms^66,73^.

In the Early-Middle Jurassic, the BNMO was rapidly extended and entered a mature stage. Deep-water facies are represented by submarine fan gravity flow deposits of the Early-Middle Jurassic Sêwa Formation. Olistostromes are generally visible in the region and locally intercalated with basalt, and ammonites and *Nereites* ichno**-**assemblages are common in sandstone and mudstone^15,67,68^. In addition, the southern margin of the South Qiangtang Basin experienced a rapid transition from a passive to an active continental margin during this period, and submarine fan deposits developed in the study area.

During the Middle Jurassic, the BNMO entered a rapid subduction stage. The South Qiangtang Basin was infilled with the Middle Jurassic Shaqiaomu Formation (J_2_*sq*) and Jiebuqu Formation (J_2_*jb*) as representative shallow marine facies. The former is mainly a mixed continental shelf facies deposition composed of clastic intercalated carbonate rocks and containing fossils such as hexahedral corals, bivalves, gastropods and crinoid stems^67,69^. The latter formation is mainly a shallow platform facies containing carbonate rocks intercalated with a small amounts of terrigenous clastic rocks that contain fossils such as hexahedral corals and bivalves^68–72,74^.

In the Late Jurassic to Early Cretaceous, the ocean closed, and a residual shallow carbonate sea became established. This is represented by the Suowa Formation (J_3_*s*) and volcanic island arc andesite rocks of the Meiriqiecuo Formation (K_1_*m*) at the edge of the basin^75–77^.

In the Late Cretaceous, the ocean completely disappeared, and the Lhasa Block collided and assimilated the South Qiangtang Basin. Subsequent terrestrial deposition include unconformable cover rocks of the Abushan Formation (K_2_*a*) or the Jingzhushan Formation (K_2_*j*), which are characterized by the piedmont molasse deposits^78–81^.

## Conclusions

Based on the study of sedimentary characteristics, sedimentary facies and paleobiostratigraphy of the Rigenco section in the Riganpeicuo area, combined with the regional geology, the following conclusions can be drawn:

1. Bivalve fossils have been discovered for the first time in the study area, all of which occur in calcareous mudstone olistostromes. Nine species from nine genera were identified. Extensive biostratigraphic correlation shows that the age of bivalves should be Late Triassic, so the age of this sequence must be younger than Late Triassic.
2. The analysis of the sedimentary facies sections shows that the main body of the section is a set of turbidite submarine fan deposits with rhythmic interbedding of mudstone and siltstone, and the upper part is a shallow marine deposit, which obviously does not meet the definition of the Jiebuqu Formation. In addition, there are a large number of calcareous mudstone olistostromes containing Late Triassic bivalves. Thus, the strata are formally reassigned to the Lower-Middle Jurassic Sêwa Formation and the Middle Jurassic Shaqiaomu Formation.
3. Based on the sedimentary facies analysis and previous research results, it is suggested that the opening of the middle part of the BNMO initiated in the Late Triassic, concurrent with the development of a shallow carbonate sea in the South Qiangtang Basin. During the Early-Middle Jurassic, the evolution of the ocean entered a mature stage, which there were a large-scale olistostromes generated along the basin margin. The South Qiangtang Basin is characterized by shallow-marine facies with mixed continental shelf and carbonate platform deposits. Oceanic closure with residual stage sedimentation and regional subduction-related magmatism occurred in the Late Jurassic-Early Cretaceous, followed by complete disappearance of the ocean in the Late Cretaceous, and the subsequent development of a piedmont molasse formation in the South Qiangtang Basin.
